# Reverse phase chromatographic behaviour of major components in *Capsicum Annuum* extract

**DOI:** 10.1186/1752-153X-6-146

**Published:** 2012-12-04

**Authors:** Monica Butnariu, Angela Caunii, Salomeia Putnoky

**Affiliations:** 1 Chemistry and Vegetal Biochemistry, Banat’s University of Agricultural Sciences and Veterinary Medicine from Timisoara, Calea Aradului no. 119, 300645, Timisoara, Romania; 2 Faculty of Pharmacy, “Victor Babes” University of Medicine and Pharmacy, 2A Eftimie Murgu Square, Timisoara, 300041, Romania; 3 Faculty of Medicine, Department of Hygiene, “Victor Babes” University of Medicine and Pharmacy, Bd. Victor Babes, no. 16, 300226, Timisoara, Romania

## Abstract

**Background:**

The purpose of the study is to develop a suitable analytical method in order to establish appropriate conditions for isolation and assay of the dominant compounds in extracts of *Capsicum annuum*.

**Results:**

The studies are performed with standard substances to establish the HPLC conditions for complete separation of capsaicin and dihydrocapsaicin, the two major components of interest. Because of their prevalent apolar features, reverse phase chromatographic version was approached. Systematic studies on different eluents revealed the 65% methanol–35% water mixture as a proper mobile phase providing a complete separation of the components according to analytical claims.

**Conclusions:**

The results may be of interest to develop a specific analytical procedure with advanced specificity for quantitative assay of capsaicin and dihydrocapsaicin in pharmaceutical products and in foods.

## Background

The two major components in *C*. *annum* are capsaicin (C) and dihydrocapsaicin (DHC). The only difference between them is the presence of a carbon–carbon double bond [1].

Capsaicin (8–methyl–*N*–vanillyl–6–nonenamide) has CAS No.: 404–86–4; molecular formula: C_18_H_27_NO_3_ and molecular weight: 305.42. Dihydrocapsaicin (8–Methyl–N–vanillylnonenamide/N–(4–Hydroxy–3–methoxybenzyl)–8–methylnonanamide) has CAS No.: 19408–84–5, molecular formula: C_18_H_29_NO_3_ and molecular weight: 307.43. Capsaicin subject hydrogenation turns dihidrocapsaicin [2]. Capsaicin is a capsaicinoid alkaloid with notable thermal stability retaining its activity even boiling [3]. It is only slightly soluble in water [4], but very soluble in ethanol and vegetable oils [5]. Other capsaicinoids are dihydrocapsaicin, nordihydrocapsaicin, homocapsaicin and homodihydrocapsaicin [6]. These are present in certain pharmaceutical products [7] or in foods [8]. From the multiple possibilities, the chromatographic technique with liquid mobile phase [9] performed at high pressure (HPLC) offers several advantages such as: the possibility of automating and computer processing the method [10]; advanced reproducibility, both in identifying the isolated compounds [11], and in the quantitative determinations [12]; the possibility of performing the analyses in a relatively short period of time [13]; and the non-destructive aspect of the analysis, the isolated components being subject to further studies [14]. Suitable methods of isolation and analysis should be useful for assessing the distribution of capsaicinoids in foods and in clarifying the roles of these biologically active substances in plant, diet, and medicine.

## Results

**Figure 1:**
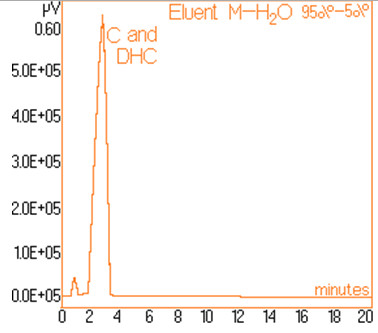
Chromatogram obtained with 95% methanol–5% water.

**Figure 2:**
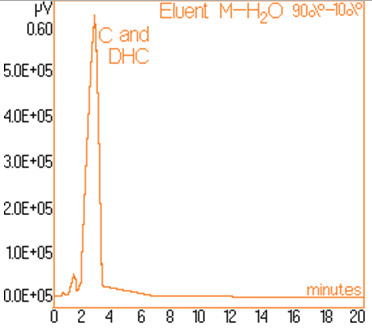
Chromatogram obtained with 90% methanol–10% water.

**Figure 3:**
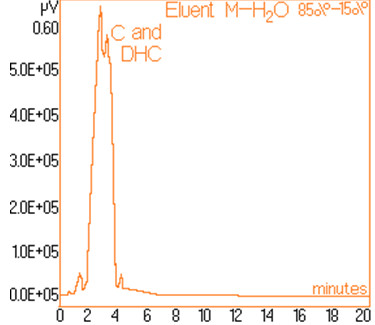
Chromatogram obtained with 85% methanol–15% water.

**Figure 4:**
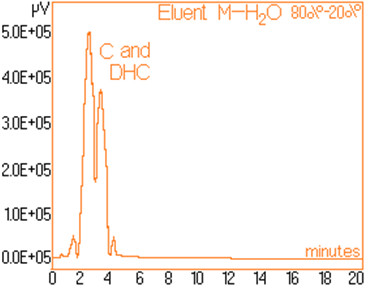
Chromatogram obtained with 80% methanol–20% water.

**Figure 5:**
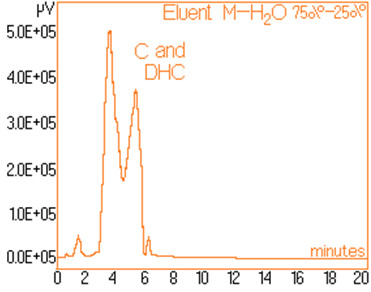
Chromatogram obtained with 75% methanol–25% water.

**Figure 6:**
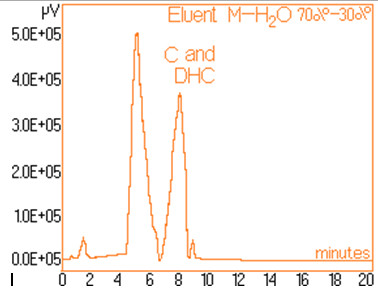
Chromatogram obtained with 70% methanol–30% water.

**Figure 7:**
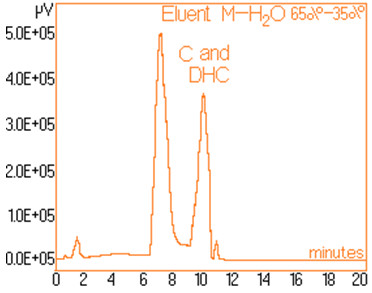
Chromatogram obtained with 65% methanol–35% water.

**Figure 8:**
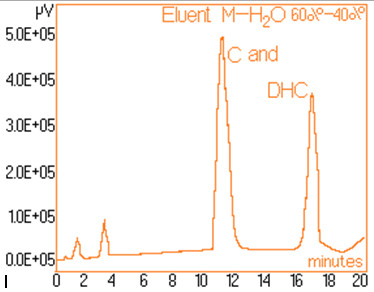
Chromatogram obtained with 60% methanol–40% water.

The standard solutions used in the series of experiments with variable eluent composition, contained 0.24 mg/mL and 0.15 mg/mL capsaicine and dihydrocapsaicine respectively. The mentioned components in the following concentrations: capsaicin and dihydrocapsaicin. The chromatograms of a standard solution containing both capsaicin and dihydrocapsaicin are presented in Figures 1, 2, 3, 4, 5, 6, 7 and 8 with indication of the mobile phase composition as well. It is found that the eluent mixture of 65% methanol and 35% water is convenient for the isolation of these components, even in complex samples. This eluent is able to completely separate the two components (“up to the base line”) without extended peak broadening. An exaggerated broadening of the peaks of interest may result in accidental overlap with foreign peaks of “ballast” components possibly present in real samples [15].

**Figure 9:**
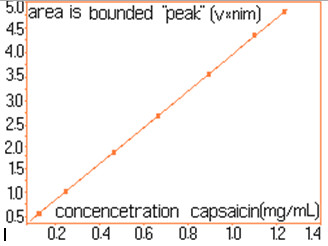
Calibration curve for capsaicin.

**Figure 10:**
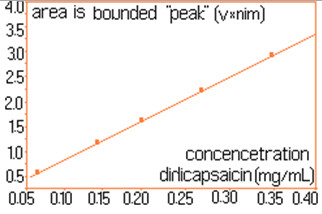
Calibration curve for dihydrocapsaicin.

**Figure 11:**
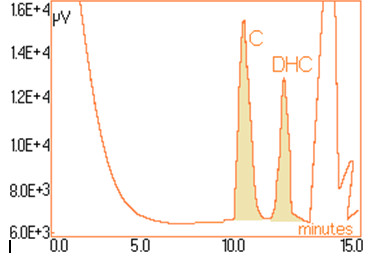
Chromatogram of C. annum extract (real sample).

**Figure 12:**
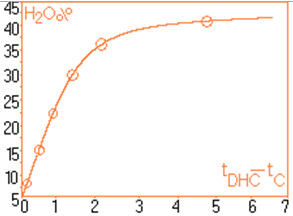
Graphic form for retention times difference for the two components, in relation to the percentage volumetric water content in eluent.

**Figure 13:**
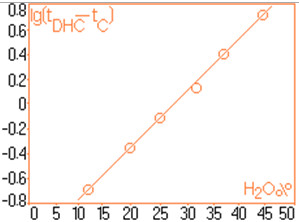
Chromatogram obtained after the injection of a diluted C. annum extract, rapport 1:10.

For quantitative determinations, calibration of the chromatographic signals (the area under the peaks) is required separately for capsaicin and dihydrocapsaicin. The details concerning the preparation of standard solutions and the associate areas of peaks are shown in Table 1. For each standard solution a volume of 20 μL was injected into the column. The calibration plot for capsaicine and dihydrocapsaicine are presented in Figures 9 and 10. The calibration plot covers the concentration interval of 0.192–1.554 mg/mL for capsaicin, with a correlation coefficient higher than 0.994, and the concentration interval of 0.055–0.444 mg/mL for dihydrocapsaicin, with a correlation coefficient higher than 0.981. The real samples (*C*. *annuum*) were previously diluted with anhydrous methanol in volumetric ratio of 1:10, and then injected a volume of 20 mL into the chromatographic column. Figure 11 presents the relevant portion of the chromatogram obtained. Figure 12 shows dependence of the retention time’s difference for the two components, to the volumetric percentage of water in eluent. The points align on a curve having a relatively simple form and monotone variation. Figure 13 shows the linearized version of the aforementioned dependence.

**Table 1:** Capsaicin and Dihydrocapsaicin standard solutions data

**No.**	**Conc. C (mg/mL)**	**Conc. DHC (mg/mL)**	**Aria C (μV**^**.**^**min)**	**Aria DHC (μV**^**.**^**min)**
1	0.192	0.055	713888.5	456893.8
2	0.388	0.111	1442649.6	923306.2
3	0.777	0.222	2889017.4	1848992.1
4	1.165	0.333	4331667.0	2772298.3
5	1.554	0.444	5778034.8	3697984.2

## Discussion

The chromatograms of Figures 1, 2, 3, 4, 5, 6, 7 and 8 shows a monotone increase of retention times both for capsaicin and dihydrocapsaicin with the volumetric percentage of water in the eluent mixture. The correlation of these variables is illustrated in Figure 12.

According to known studies and theories concerning the chromatographic migration in reverse phase chromatographic systems [16], the logarithm of retention times are in linear relationship with the polarity of the mobile phase (Figure 13). The found linear relationship demonstrates that in the retention mechanism of capsaicin and dihydrocapsaicin, prevails the same type of interaction (the hydrophobic interaction).

Taking into account the practical aspects [17] for relevant analyses, there were selected the conditions in which the chromatograms from Figures 1, 2, 3, 4, 5, 6, 7 and 8 were obtained.

In Figure 11 it is observed that in the chromatogram of real samples (*C*. *annuum*), besides the chromatographic signals of interested components, there appear, with impressive intensities, other peaks belonging to “ballast” components. However, the interested signals are visible and can be integrated without difficulties [18]. Table 2 contains the results of analyses performed on a series of 16 *C*. *annuum* extracts, randomly chosen from different batches but from the same producer.

**Table 2:** **Content of capsaicin and dihydrocapsaicin in *C. annuum***

**No.**	**Aria C (μV**^**.**^**min)**	**C in extract (mg/mL)**	**Aria DHC (μV**^**.**^**min)**	**DHC in extract (mg/mL)**
1	2544217.6	6.843	1322642.7	1.578
2	2583310.2	6.948	1364409.0	1.637
3	2338947.5	6.290	1277005.4	1.533
4	2511509.0	6.755	1320163.7	1.584
5	2462441.1	6.623	1255441.2	1.507
6	2481150.2	6.673	1266714.0	1.520
7	2396704.7	6.446	1275194.6	1.530
8	2555477.2	6.873	1334765.8	1.602
9	2468812.0	6.640	1253386.6	1.504
10	2511900.4	6.756	1288022.2	1.546
11	2356115.0	6.337	1235534.9	1.483
12	2505579.7	6.739	1257196.3	1.509
13	2331576.0	6.271	1143057.2	1.372
14	2407052	6.474	1253863.8	1.505
15	2298857.3	6.183	1239700.5	1.488
16	2395154.3	6.442	1140557.8	1.369
Values mean			6.581	1.517
Standard deviation			0.236	0.071

It is noticed that the individual values of concentrations, for both components, oscillate within relatively close limits, proving that the 16 extracts from different samples of *C*. *annuum*, although coming from different batches, had resembling compositions. In this situation, by adequately modifying the eluent composition [19], we can reach a similar situation to that presented in Figure 12, situation in which the signals of the two components can be evaluated conveniently.

## Conclusions

This assay represents a method for the isolation and the quantitative analysis of secondary capsaicinoid metabolites (capsaicin and dihydrocapsaicin), in 16 fruit samples of the *Capsicum* (*Solanaceae*) plant was investigated. The HPLC method was optimized by highlighting the effects on retention times of the composition of the mobile phase (methanol and water mixture). Based on the area defined by the signals of components (shown in columns 2 and 4, in Table 2) and with the calibration lines build with known standard solutions (Figures 9 and 10), there were determined the concentrations of the components in the solutions of real samples. Obviously, the concentrations in the original samples were 10 times higher (because of the dilution), than the ones read directly from Figures 9 and 10. The concentrations of capsaicin and dihydrocapsaicin, expressed in mg/mL, in extracts from *C*. *annuum*, are presented in the columns 3 and 5, in Table 2. The described and successfully tested analytical method, in the case of *C*. *annuum* allows, with little modifications if needed, the isolation and assay of the two components even in presence of a large quantity of “ballast” components.

In conclusion, it been shown that the nonpolar stationary phase and a mobile phase composed of 65% methanol and 35% water is suitable for a good chromatographic separation and a convenient assay of capsaicinoids (capsaicin and dihydrocapsaicin).

## Methods

### Plant material

The fruits of *C*. *annuum* were collected from the local laboratory cultures in western Romania. The samples used for assays were retained, numbered and stored in the Biochemistry plant refrigerator. For the preparation of the eluent mixture, anhydrous methanol (Merck, spectroscopic purity “Uvasol”) and standard solutions of capsaicin and dihydrocapsaicin of the same brand were used.

### Capsaicinoid isolation

Prior to grinding, bags containing pod samples from each of the 16 samples were taken out of the freezer and allowed to reach room temperature prior to opening, in order to prevent moisture condensation onto the pod surface. Representative fruits were ground with a GM 200 (Grindomix) mill to pass a 20 screen mesh, not more than a week prior to capsaicinoid isolation. The powder (0.200 g) was accurately weighed into 2 vials, 2 mL of N, N–dimethylformamide (DMF) was added and the vial was sealed with Teflon-lined screw caps. Extraction was carried out at 80°C in a dry block heater for 1 h. Samples were swirled every 15 min during the 1 h period to assure proper mixing. Samples were then removed from the heat block, centrifuged for 5 min in a Rotofix 32 A EC 34.1 centrifuge and supernatants were decanted into a 10 mL volumetric flask. The procedure was repeated three more times for a total of 4 extractions and the content of flask were brought to volume with DMF. Preliminary recovery trials were conducted to establish capsaicin (C) and dihydrocapsaicin (DHC) recoveries of 95% and 101%, respectively.

### Capsaicinoid analysis for high performance liquid chromatography (HPLC)

Samples of the extracts of *C*. *annuum* were placed into autosampler vials and used directly for HPLC analysis using a modification of the “short run” procedure of Morales [20]. The chromatograms were performed with a unit from Jasco, equipped with a programmable pump unit (PV–980 type), UV–970 type optical absorption detector having continuously adjustable wavelength between 190–900 nm, programmable agitator type LG–980–0.2S and a DG–1580–54 type degassing unit.

Separations were accomplished with a C18 NUCLEOSIL 100 column, filled with particles with 5 μm diameter. The samples used in the testing phase were fragments of *C*. *annuum* extract obtained randomly from different batches. In order to establish the appropriate eluent composition for separation of the two components under discussion, and for their isolation from other components present in the sample, dedicated studies were reported [21], concerning the chromatographic migration of capsaicin and dihydrocapsaicin at progressively increasing polarity of the mobile phase [22]. In all determinations methanol–water mixtures of different compositions were used. The polarity of eluent was related to the volumetric percentage of water in the mixture. The components leaving the column were monitored at 220 nm [23]. The eluent flow throughout all the experimental series was of 1 mL/minute.

## Competing interests

The authors declare that they have no competing interests.

## Authors' contributions

MB conceived the study, participated in the design and co-ordination of the experiments and data interpretation and helped draft the manuscript. AC and SP produced samples, performed data analysis and data interpretation. All authors have equal rights and all authors read and approved the final manuscript.
